# The Promises and Challenges of Ecological Momentary Assessment in Schizophrenia: Development of an Initial Experimental Protocol

**DOI:** 10.3390/healthcare3030556

**Published:** 2015-07-15

**Authors:** Brandon A. Gaudiano, Ethan Moitra, Stacy Ellenberg, Michael F. Armey

**Affiliations:** 1 Butler Hospital and Warren Alpert Medical School of Brown University, 345 Blackstone Blvd., Providence, RI 02906, USA; E-Mails: stacyellenberg@gmail.com (S.E.); michaelarmey@gmail.com (M.F.A.); 2 Warren Alpert Medical School of Brown University, Box G-BH, Providence, RI 02912, USA; E-Mail: Ethan_Moitra@brown.edu

**Keywords:** schizophrenia, severe mental illness, psychosis, treatment adherence, ecological momentary assessment, experience sampling methods, mobile technology, smartphones, psychological assessment, psychiatric hospitalization

## Abstract

Severe mental illnesses, including schizophrenia and other psychotic-spectrum disorders, are a major cause of disability worldwide. Although efficacious pharmacological and psychosocial interventions have been developed for treating patients with schizophrenia, relapse rates are high and long-term recovery remains elusive for many individuals. Furthermore, little is still known about the underlying mechanisms of these illnesses. Thus, there is an urgent need to better understand the contextual factors that contribute to psychosis so that they can be better targeted in future interventions. Ecological Momentary Assessment (EMA) is a dynamic procedure that permits the measurement of variables in natural settings in real-time through the use of brief assessments delivered via mobile electronic devices (*i.e.*, smartphones). One advantage of EMA is that it is less subject to retrospective memory biases and highly sensitive to fluctuating environmental factors. In the current article, we describe the research-to-date using EMA to better understand fluctuating symptoms and functioning in patients with schizophrenia and other psychotic disorders and potential applications to treatment. In addition, we describe a novel EMA protocol that we have been employing to study the outcomes of patients with schizophrenia following a hospital discharge. We also report the lessons we have learned thus far using EMA methods in this challenging clinical population.

## 1. Introduction and Background

### 1.1. Treatment Nonadherence Is a Major Public Health Problem in Schizophrenia

Patients with psychosis are at an increased risk for treatment nonadherence, which is a costly problem. Schizophrenia is among the top ten causes of disability worldwide [1], with estimated treatment costs of $62.7 billion per year [2]. Furthermore, research indicates that 50% of patients with schizophrenia are medication nonadherent [3]. Nonadherence is predictive of poorer outcomes, including relapse, rehospitalization, self/other harm, and homelessness [4]. It is estimated that improved adherence could save over $100 million per year in inpatient costs for Medicare alone [5]. In addition to medication nonadherence, poor behavioral adherence (e.g., missed appointments, drop out) to other treatments (e.g., psychotherapy, case management) also puts patients at high risk for negative outcomes such as relapse and hospitalization [6].

The post-hospital period is a critical time to study adherence because it is particularly prevalent and problematic after discharge. Medication nonadherence, session nonattendance, and drop out increase risk for rehospitalizations. In addition, recent hospitalization predicts reduced adherence [7,8,9] and the transition from inpatient to outpatient treatment produces the highest risk of nonadherence and drop out [6,10]. Recently hospitalized patients are adjusting to new medications, dosages, and new outpatient treatments. Cost, time, and other barriers limit the ability of clinicians to monitor successful transition from inpatient to outpatient care, leading to adherence problems.

### 1.2. Our Knowledge of Adherence Predictors and Interventions Is Limited

Research has identified a number of different adherence predictors [11]. However, little is known about the relative contribution of these variables when combined, and even less is known about how these barriers fluctuate in the patient’s natural environment. Prior studies are mostly cross-sectional/retrospective in nature [11], and few have examined factors that influence day-to-day adherence. Retrospective assessment is problematic because memory bias may influence self-reporting [12]. In addition, many adherence barriers fluctuate according to environmental circumstances and mood states, making them difficult to assess out of context [13,14]. Previous retrospective research suggests that treatment complexity [15,16]; patient-provider alliance [17,18]; treatment attitudes/beliefs [19,20]; cost/access [21]; and medication side effects [19,22] are important to consider in adherence to treatments. Situationally-cued factors such as self-stigma [17,23], environmental stressors and inadequate support system [24], forgetfulness [25], and reduced insight [26,27,28] predispose patients to nonadherence. Lastly, psychological symptoms and their expression are predictive of adherence, with situationally varying emotional states [11], substance use [11], and positive symptoms [29] predicting nonadherence.

Traditional psychosocial adherence interventions for schizophrenia have produced mixed results to date [4,30,31]. Psychoeducational interventions alone generally have been found to be ineffective for improving adherence in schizophrenia [31]. Compliance therapy (with cognitive-behavioral and motivational strategies) showed promise for reducing nonadherence in an initial trial [32], but there was a subsequent failure to replicate findings [33]. A recently developed treatment that combines motivational and behavioral strategies tailored to target different problems related to nonadherence produced significantly improved service engagement and medication adherence compared with treatment as usual in patients with psychotic disorders without substance abuse [3]. Other behavioral and family approaches have shown promise in initial studies [4,30,31]. However, the most recent Patient Outcomes Research Team guidelines concluded: “there is insufficient evidence to recommend any specific intervention to promote adherence to antipsychotic medications among persons with schizophrenia” [34] p. 61. Without advancing the field’s knowledge of adherence barriers, there is little hope for improving the effectiveness of adherence interventions. Furthermore, few adherence studies have addressed the issue of behavioral adherence, as it differs from medication adherence [35]. Behavioral forms of adherence (e.g., appointment attendance) are critical in maintaining treatment gains, preventing relapse [36], and improving medication adherence [37]. Research is needed to examine adherence predictors *in vivo* to identify dynamic fluctuations and the influences of situational/contextual factors to improve prediction of adherence behaviors.

### 1.3. Ecological Momentary Assessment (EMA) Can Improve Our Understanding of Nonadherence

Newer technologies can be used to develop a more ecologically-valid understanding of adherence. The measurement of adherence barriers, including social support, stressors, substance use, affect, and psychotic symptoms, is best suited to an approach that is sensitive to dynamic changes in adherence behaviors in context. This can be provided through real-time assessment using experience sampling or ecological momentary assessment (EMA) [38]. Mobile devices and other personal digital assistants have become the ideal technological platform for collecting *in vivo* data. EMA has been shown to be reliable and valid for measuring adherence in several studies, but, to date, mostly in pediatric populations (e.g., cystic fibrosis) [39,40].

Preliminary research supports the short-term feasibility and acceptability of EMA in psychosis via mobile devices [41,42]. Various studies show that about 90% of people with schizophrenia accept and can be trained to use EMA and that compliance is comparable and sometimes superior to that of nonclinical populations (completing at least 75% of assessments) [25,42,43,44,45]. Most importantly, EMA has demonstrated incremental validity over traditional retrospective reports in assessing symptoms associated with schizophrenia, with one study finding that spikes in daily paranoia were captured via EMA in patients who reported low paranoia in traditional, retrospective assessments [46]. Another study demonstrated the incremental validity of EMA compared to retrospective symptom reports in individuals with schizophrenia compared to healthy controls [43]. In addition, Oorschot *et al.* [47] found inconsistent reporting of symptomatology between ecological assessments and clinical assessments on measures of delusions and auditory hallucinations, but not on measures of suspiciousness and visual hallucinations, highlighting the importance of capturing a moment-to-moment assessment of specific symptoms.

Furthermore, EMA can be used to identify factors that could lead to refinements in psychosocial interventions for schizophrenia. Ecological momentary interventions provide moment-to-moment self-management techniques to patients, cued by triggers from ecological assessments. One pilot study showed the feasibility and acceptability of using ecological momentary intervention via mobile devices with schizophrenic patients, and demonstrated low dropout rates and high satisfaction [48]. Montes *et al.* [49] used short message service (SMS) to provide outpatients with schizophrenia with daily medication reminders for three months. As a result, adherence and attitudes towards medications were both significantly improved as compared with the control group. Furthermore, improvements were seen in cognitive, global, and negative symptoms. The current project builds on this work by expanding and improving assessment of adherence barriers and using data to identify contextual modifiable predictors to target in future interventions.

### 1.4. Rationale and Hypotheses for Current Project

Our study aims to advance the field’s understanding of factors that can limit the impact of treatments due to nonadherence via the assessment of adherence barriers in the patient’s natural environment. Patients with psychosis often require long-term adherence to medications and other outpatient therapies, most critically upon discharge from the hospital. Reduced adherence to these treatments and premature drop-out both put individuals at high risk for acute hospitalization and other negative outcomes. Previous research has examined a number of risk factors for nonadherence, but little is known about how these variables fluctuate in the moment that can be better targeted by future self-management interventions. The use of new technologies can lead to an improved understanding of the factors that place patients at high risk for nonadherence and acute hospitalization. EMA is the ideal approach to fill this research gap. Initial steps have been taken to demonstrate that EMA is a feasible and acceptable assessment procedure for community-dwelling patients. However, we are not aware of previous EMA studies in patients with schizophrenia during the critical period from inpatient to outpatient treatment, which may have unique risk factors. The next step is to further refine EMA specifically focused on post-hospital adherence to guide future technology-assisted intervention development.

In the present study, our primary outcomes of interest will be (a) medication adherence, based on a multi-modal assessment using electronic monitoring, pill counts, and self-report; and, (b) behavioral adherence to outpatient treatments, measured by self-report of treatment utilization. We hypothesize that nonadherence to either outcome will be predicted by several dynamic ecologically assessed variables: (a) occurrence of positive psychotic symptoms; (b) negative affect; (c) occurrence of stressful life events; (d) lack of social support; (e) experience of medication side effects; (f) poor working alliance with the treatment provider(s); (g) negative medication/treatment beliefs; (h) substance use; and (i) avoidance-based psychological coping.

## 2. EMA Study Procedures

### 2.1. Sample Selection

Participants in this study will consist of 60 patients recruited during a psychiatric hospitalization. We used Zhang and Wang’s simulation procedure to estimate our needed sample size based on our main outcomes of medication and behavioral adherence [50]. Our simulation assumed seven repeated assessments for EMA data, a moderate correlation between slopes and intercepts (*r* = 0.30), a total missing data rate of ~20%, and 2500 simulation replications per sample size. The proposed sample of participants should be adequate (Power = 0.80) to detect a medium (clinically significant) effect (Cohen’s d = 0.425) for the full sample. Based on our previous experience with this population, we anticipate <15% study attrition. We plan to recruit additional patients to compensate for potential drop-out where possible. Inclusion criteria are: (1) currently hospitalized; (2) DSM-5 criteria for a psychotic-spectrum disorder (*i.e.*, schizophrenia, schizoaffective disorder, delusional disorder, schizophreniform disorder, psychosis NOS) or a mood disorder (major depression or bipolar disorder) with psychotic features based on diagnostic interview (Structured Clinical Interview for DSM); (3) 18 years or older; (4) prescribed oral antipsychotic medication; and (5) ability to speak and read English sufficiently to complete the assessments. Exclusion criteria are: (1) planned discharge to supervised living settings or participation in outpatient adherence programs (e.g., medication packaging/monitoring); (2) pregnancy or medical condition contraindicating use of antipsychotics (e.g., dementia); or (3) homelessness.

We chose to expand our recruitment to include patients with psychotic mood disorders for several reasons. First, we were interested more broadly in studying the interactions among positive psychotic symptoms, mood symptoms, and treatment adherence across different levels of severity and comorbidities. Second, we were recruiting patients from an inpatient setting where initial diagnosis can be unclear and patients often transition from psychotic mood disorders to primary psychotic disorders over time. Thus, we were hoping to be able to “catch” this process as it develops in some patients. Third, EMA has widely been studied in patients with primary mood disorders [51], but not those with mood disorders with psychotic features. However, we believe that the predictors of nonadherence may be very similar across groups. Furthermore, in the current study, it is important to note that current or past risk for self-harm/suicidality and harm to others are assessed at intake but are not exclusion criteria. As needed, licensed study clinicians conduct safety assessments and provide appropriate intervention (e.g., referral to emergency services, community treatments resources) for patients indicating risk at the baseline and follow-up assessments.

### 2.2. Feasibility of EMA

We assessed the acceptability/feasibility of our EMA protocol in five inpatients with schizophrenia at our recruitment site. Patients were trained to use the device and complete the EMA assessment. We collected data from four patients who reported willingness to use the device. They reported (scale = 1–5) that they liked using the device (M = 4; SD = 1) and it was understandable (M = 5; SD = 0), interesting (M = 4; SD = 1.4), and respectful (M = 4.75; SD = 0.5). We obtained feedback from one patient with delusions about technology unwilling to use device but willing to answer the assessment questions in another form (e.g., diaries) and found them acceptable. We used this information to further refine our protocol.

### 2.3. Choice of EMA Variables

Treatment adherence is multi-determined. We chose to employ a “risk reduction” model similar to those utilized in heart disease and diabetes [52,53,54,55]. Modifying risk factors is hypothesized to decrease the incidence of unwanted outcomes. Some known predictors of nonadherence are relatively static and difficult or impossible to change (e.g., demographics, treatment access/costs). These variables are not assessed via EMA. For EMA, we selected targets that are: (a) potentially malleable, (b) related to adherence, and (c) could be impacted by future intervention strategies. Data suggest the following variables are relevant to EMA, given their dynamic nature and relationship with adherence: positive psychotic symptoms [29]; affect [11]; daily stressors [24]; social support [24]; side effects [25]; working alliance with the treatment provider [17,18]; negative medication/treatment beliefs [20,56]; substance use [11]; and psychological coping [57]. See Table 1 for description of EMA protocol and sample questions. When possible, we selected items used successfully in previous EMA research (see Table 1 for references). When this was not possible, we adapted EMA items from reliable and valid measures that were otherwise too long for EMA administration. This is a common strategy in EMA research, which often requires modifications to psychometric assumptions given the frequency and brevity of these assessments compared with traditional paper-and-pencil measures [58,59]. In other words, the primary aim of EMA involves a tailored measurement approach that is adapted specifically to the target population and goals of the assessment.

**Table 1:** Ecological Momentary Assessment (EMA) Protocol.

Schedule	Dynamic Variables	Sample Items
Random Assessment	*Context*	Where are you right now? [42]
	*Substance Use*	Have you used any of the following substances?
	*Affect*	Rate the following words based on how you feel right now [60].
	*Psychotic Symptoms*	Was someone spying on you or plotting against you? [42]
	*Psychological Coping*	How did you deal with any difficulties you were having? [61]
	*Psychological Acceptance*	I simply noticed my feelings and continued with what I was doing [62].
	*Stressors*	Has something stressful or bad happened in your life? [63]
	*Social Support*	I am getting the emotional help and support I need from my family or friends [64].
End of Day Assessment	*Behavioral Adherence*	Did you attend any treatment appointments today?
	*Medication Adherence*	I’ve taken my psychiatric medications as prescribed today.
	*Therapeutic Alliance*	Do you and your treatment provider understand each other? [65]
	*Reasons for Nonadherence*	My medications make me feel strange or “doped up.” [66,67,68,69]
	*Medication Side Effects*	How bothered were you by these side effects? [70]

We propose to understand adherence behaviors within a *contextual behavioral science* (CBS) framework, which is the basis of Acceptance and Commitment Therapy (ACT), a newer cognitive-behavioral approach that has empirical support for treating psychosis [57,71,72]. CBS is an inductive, bottom up approach that emphasizes intervention development that is tightly linked to principles of behavior change derived from basic research [73]. A contextual approach “views psychological events as ongoing actions of the whole organism interacting in and with historically and situationally defined contexts” (p. 4) [74]. This framework suggests that an individual’s behavior change is dependent upon the dynamically changing internal and external environment and that detrimental behaviors (*i.e.*, nonadherence) occur most often when individuals are attempting to avoid discomfort, despite how avoidance can negatively impact them. EMA is the ideal way to model this framework. We believe that our proposed EMA variables would be potentially modifiable based on future mobile interventions [75] based on CBS and ACT. A mobile intervention is a “therapist in your pocket” and can provide self-management skills by delivering algorithm-based, personalized coping strategies to target adherence barriers.

### 2.4. EMA Procedures

We have been piloting a version of our EMA protocol using the open source, *MyExperience Tool* [76] (http://myexperience.sourceforge.net/) software package to be administered via Windows-compatible mobile devices. EMA consists of: (1) *Random schedule*: allows for daytime hours (9 am–9 pm) to be split into three, 4-h segments, scheduling a random sample in each of these three segments. Each assessment takes 5–10 min. Each assessment is at least 30 min removed from a prior/subsequent assessment. Random assessments focus on variables that are likely to fluctuate throughout the day, including substance use, affect, psychotic symptoms, coping with symptoms, and experiences of environmental stressors. (2) *End of Day schedule*: Appointment attendance with mental health providers (*i.e.*, psychiatrist, nurse, social worker, and therapist), working alliance, and reasons for nonadherence are assessed here. We also specifically assess medication adherence and experiences of medication side effects at the end of each day. All participants undergo an initial EMA training with research staff, in which they practice responding to EMA and familiarize themselves with the device. To ensure that participants who are highly symptomatic or cognitively impaired are able to successfully complete EMA, participants are instructed to contact us if questions arise during their use of the mobile device. On a weekly basis, a study staff person calls the patient and offers to answer questions or respond to any technical issues that might be interfering with his/her ability to respond to the EMA assessments. The contacts are kept as brief as possible (*i.e.*, typically <5 min). As a backup plan, it is possible to consider replacing EMA with paper/pencil diaries in rare instances that remedial training does not resolve these issues.

### 2.5. Traditional (Non-EMA) Assessments

In addition to EMA-administered questions, we also use retrospective study measures completed via traditional pen-and-pencil methods. These are administered at baseline, 1- (*i.e.*, the conclusion of the EMA phase), two-, and four-month follow-ups and are intended to complement our EMA battery. We also assess acceptability and potential burden of our assessment strategy. Additional study measures are listed in Table 2.

We use the *Medication Event Monitoring System (MEMS)* to assess adherence to the primary antipsychotic medication post-hospital discharge. MEMS uses electronic pill caps that record bottle openings/closings and the corresponding time/date. Electronic monitoring is recommended for objective assessment [77]; MEMS has been used in past schizophrenia studies with success [78]. We follow published guidelines for training patients to use MEMS and for analyzing data ([79,80]). Patients are instructed to bring their prescribed antipsychotic medication to our clinic and are trained by study staff to use MEMS at the start of the study. The research assistant demonstrates the use of MEMS in front of the patient and answers any questions the patient may have. Then, the patient is asked to transfer his/her medication to MEMS and label it according to the instructions on the original prescription. The research assistant supervises the process to ensure that the patient is using MEMS correctly. In the event that the patient is unable to return to our clinic to complete the MEMS (e.g., medication refills occurring between assessments or difficulty returning due to problems accessing transportation), the staff person assists the patient in using the MEMS system by providing additional instruction over the telephone as needed. Using MEMS is similar to patients using other pill organizer systems. Patients return to our clinic at one-, two-, and four-month assessments so that we can download the MEMS data from the cap. The patients are instructed to call the staff person on the number provided in the event of medication changes between visits, problems using MEMS, or any questions that may arise about using the caps during the study.

**Table 2:** Traditional (Non-EMA) Study Measures.

Measure	Topic	Format	Time Point
Treatment History Interview-4 (THI-4) [81]	Treatment Utilization/ Behavioral Adherence	Interview	BL, 1, 2, 4
Medication Event Monitoring System (MEMS) [77]	Medication Adherence	Electronic	BL, 1, 2, 4
Pill Counts [82]	Medication Adherence	Behavioral	BL, 1, 2, 4
Brief Adherence Rating Scale (BARS) [78]	Medication Adherence	Interview	BL, 1, 2, 4
Ratings of Medication Influences Scale (ROMI) [69]	Reasons for Nonadherence	Interview	BL, 1, 2, 4
Drug Attitude Inventory-10 (DAI-10) [68]	Medication Attitudes	Self-Report	BL, 1, 2, 4
Antipsychotic Side Effect Checklist (ASC) [83]	Medication Side Effects	Interview	BL, 1, 2, 4
Working Alliance Inventory-Short Version (WAI-S) [65]	Doctor-Patient Therapeutic Alliance	Self-Report	BL, 1, 2, 4
Brief Psychiatric Rating Scale (BPRS) [84]	Psychiatric Symptoms	Interview	BL, 1, 2, 4
Positive and Negative Affect Schedule-Expanded (State) (PANAS) [60]	Mood State	Self-Report	BL, 1, 2, 4
Consumer Experiences of Stigma Questionnaire (CESQ) [67]	Stigma/Discrimination	Self-Report	BL, 1, 2, 4
Life Events Assessment (LEA) [63]	Stressful Life Events	Interview	BL, 1, 2, 4
Multidimensional Scale of Perceived Social Support (MSPSS) [64]	Perceived Social Support	Self-Report	BL, 1, 2, 4
Emotional Regulation Questionnaire (ERQ) [85]	Psychological Coping	Self-Report	BL, 1, 2, 4
Acceptance and Action Questionnaire-II (AAQ) [86]	Psychological Flexibility/ Experiential Avoidance	Self-Report	BL, 1, 2, 4
World Health Organization Disability Assessment Schedule 2.0–12 Item Version (WHODAS 2.0) [87]	Psychosocial Functioning	Self-Report	BL, 1, 2, 4
Addiction Severity Index (ASI) [88]	Illicit Drug Use	Interview	BL, 1, 2, 4
Alcohol Use Disorders Identification Test-Consumption (AUDIT-C) [89]	Hazardous Drinking	Self-Report	BL, 1, 2, 4

*Note*. Assessment time points: baseline (BL), 1, 2, and 4 months post-hospitalization.

Some patients do not find MEMS acceptable and refuse to use it. In addition, patients may be unable to use MEMS depending on the method used to take medications (e.g., use of pill containers or blister packs). Therefore, we use pill counts as an alternative objective measure of adherence when MEMS is not available. We follow similar procedures as those described by Kalichman *et al.* [82]. In brief, patients are instructed to: (a) bring their primary antipsychotic medication being assessed for adherence to the assessment visit, (b) provide the medication bottle with prescription information for the research assistant to record, (c) report lost or gained pills since the previous count, (d) place the pills in the bottle on a disposable plate for counting, (e) count the pills on the plate, (f) double count the pills for accuracy, and (g) then replace the pills in the original bottle.

### 2.6. EMA Data Analysis Strategy

**Figure 1:**
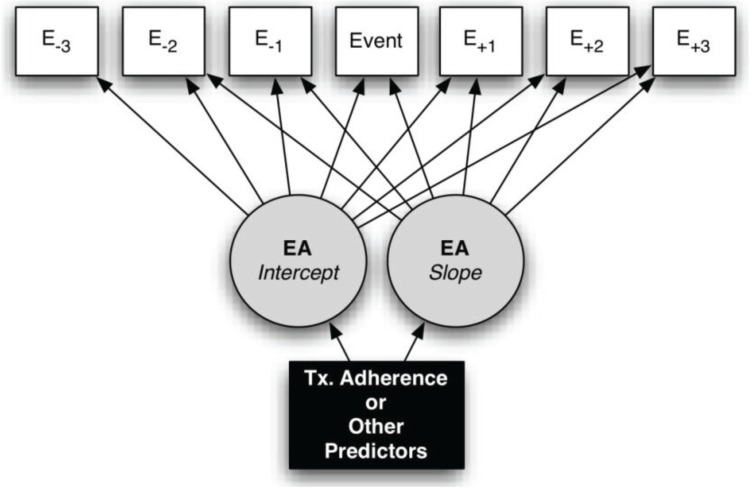
Model for one ecological assessment (EA) variable (e.g., psychotic symptoms) as a dynamic predictor of treatment adherence. “E” reflects nonadherence event, with + and − denoting assessments before or after event.

EMA produces large amounts of data and requires different strategies for analysis compared with traditional (less frequent) longitudinal assessment. We will initially explore the utility of EMA measures by identifying episodes of nonadherence. Using that point as an anchor, we will identify three ecological assessments prior to (E-3, E-2, E-1) and immediately following (E + 1, E + 2, E + 3) the nonadherence event (E). We will also randomly identify an adherence event and select three ecological assessment points before and after this adherence event as a comparison. We will then directly compare incidents of adherence to nonadherence using the dynamic adherence constructs identified above. Using latent growth curve modeling (LGCM; see Figure 1), we will estimate intercept (*i.e.*, mean) and slope (*i.e.*, linear rate of increase/decrease) for each construct, with the option to explore nonlinear outcomes (*i.e.*, quadratic) should linear slope provide a poor fit to the data. We will fix our initial two time points, and estimate remaining time parameters. We will initially fit unconditional models to determine optimal change trajectories for each outcome, with useful measures of nonadherence evidencing a differing slope or intercept than adherence events. Following specification of LGCM, we then fit a series of iterative conditional growth models in which our parameters are regressed on selected predictor variables, allowing for the retention/rejection of each variable and the incremental utility of adding ecological adherence measures to traditional assessments.

### 2.7. Future Ecological Momentary Intervention Development

After identifying the relative importance of our adherence predictors and the degree to which these variables change, we will be well-poised to draw upon our treatment expertise to develop a mobile device-based adherence intervention for a future project using an ecological momentary intervention approach. We will use our EMA protocol as a model, appending assessment of variables found to be salient in this study with brief interventions matched to modify their influence on adherence. For instance, if we find that psychological coping with stressors is particularly impactful on daily adherence, we will use EMA to probe for stressors when they occur, and automatically respond with a brief intervention to prompt appropriate coping strategies (e.g., cognitive, behavioral, motivational). Depending on device connectivity, it would be possible to electronically communicate nonadherence to providers in (or near) real-time, perhaps triggering telephone outreach by clinical staff to address patients’ concerns or other variables undermining adherence. An immediate response (and potential resolution) could prevent decompensation and reduce the risk of rehospitalization. Thus, our future intervention will be personalized to the individual and his/her daily experiences. Also, ecological momentary interventions have the potential to be highly disseminable once developed because they can have greater reach than traditional intervention approaches relying on individual therapists for delivery [90]. Once our mobile intervention is initially developed, we plan to test it out in community mental health settings to explore how it can be integrated into existing treatment programs to complement standard treatments and provide greater support to patients at home. We plan to solicit feedback from patients and providers about the acceptability and usability of our mobile intervention and to continue to develop it in an iterative fashion. This will ensure that our mobile intervention is being developed in ways that will prove ultimately usable by consumers and workable within current systems of care, following similar models used by other investigators [91].

## 3. Lessons Learned Thus Far

### 3.1. Choice of Mobile Devices and Programming

Successful EMA research depends on reliable hardware and software. We used the *MyExperience Tool* [76] to develop our EMA paradigm. *MyExperience* is an open-source, freely available software package that facilitates real-time data collection on mobile devices using XML coding. It runs on the Windows Mobile operating system, meaning that we were limited to Windows devices, rather than iOS (Apple, Cupertino, CA, USA) or Android-driven operating systems. We chose *MyExperience* because it is free, reliable, and simple. However, it should be noted that from the development of this study two years ago to the present, *MyExperience* has become somewhat outdated. This is common in the software industry as innovations in technology and programming move at a rapid pace. Newer software, such as Mobile EMA (mEMA; Rensen St. Lansing, MI, USA) or Apple’s new ResearchKit (Apple, Inc., Cupertino, CA, USA), can improve programming capabilities using more advanced features and provide more flexible data collection opportunities.

A recent Pew Center study showed that 88% of Americans over the age of 18 have a cellular phone, smartphone or otherwise [92]. Although we expected that most of our participants would have a mobile device, we could not guarantee their uniformity or reliability. Therefore, we chose to provide devices to our participants to standardize the delivery platform and ensure control over the software. Because of concerns that participants might damage or lose the devices we provided, plus potential risk of participants selling the devices, we sought devices that were affordable and somewhat “outdated,” meaning that their resale value was less than the potential monetary incentives we would provide for completion of EMA and returning the device to us.

As such, all participants were given older-generation Palm mobile phones. These were somewhat less user-friendly than newer smartphones (e.g., less responsive touch screen), but their benefits outweighed the costs of a more expensive, newer device. Another issue we ran into is that many mobile devices will only boot with a SIM card; because we did not enable the Palm devices with telephone or wireless service, we used “dummy” SIM cards to facilitate successful operation. The Palm devices and SIMs were purchased from the secondary market, making their availability more variable. The devices were enabled to contact emergency services (911). Although it is possible to transmit data from mobile devices to a research database, we chose not to enable wireless access as our funds were limited and our paradigm did not require real-time communication between patients and researchers. Instead, we incentivized patients $50 to return the devices to us, further reducing the risk of loss or resale.

### 3.2. Recruitment Strategies

We plan to recruit 60 adult patients referred from psychiatric units at our hospital. Initially we targeted inpatients only. However, over the course of our study, we expanded recruitment to the partial hospitalization program to ensure that we met recruitment goals and also to assess a wider range of functioning and symptoms in our sample. Our experience showed that partial hospital patients were generally better functioning than inpatients despite often having clinically significant psychotic symptoms.

A research assistant reviews new admissions via an electronic medical records system and obtains physician permission to approach the potential participant about the study. We learned that successful recruitment depends on using a collaborative recruitment approach and being sensitive to the patient’s clinical status at the time of recruitment. Because our study is assessment-only, we do not offer treatment to potential participants. In addition to being motivated to participate because of the compensation provided, we realized patients would be more willing to participate if they believed that their participation could contribute to the greater good. When approaching potential participants, the research assistant describes the study and emphasizes how disruptive and unpleasant the hospitalization process can be for many patients. The research assistant highlights the need to better understand what leads to hospitalization, noting our goal of helping people stay out of the hospital when possible. The priority is to engage the patient by acknowledging how he/she can help other patients. Because EMA is novel to most patients, the research assistant demonstrates how the study works on a device during the recruitment conversation. This shows how our study is different and perhaps more enjoyable than traditional pencil-and-paper assessment studies.

Given the nature of our target population, it is common for patients to be too sedated due to medication or too symptomatic to be fully attentive and receptive to the recruitment conversation at first. The research assistant works closely with hospital staff to determine the appropriate time to make first contact with patients, to recognize when they cannot make an informed consent decision, and to determine when to re-approach the patient. Every patient has the right to refuse participation, but we do not want them to make this decision without their full cognitive capacity.

### 3.3. Retention Strategies

We compensate participants for completion of the one-month EMA phase and returning the device ($50) and each two- and four-month in person follow-up assessment ($25). Patients are also incentivized $0.50 for each of the 120 EMA assessments completed for up to an additional $60 in compensation. Further, we maintain flexibility in scheduling and accommodating patients to encourage retention. As needed, we provide transportation to and from our research site and if needed, will conduct home visits to recover the device and complete assessments. Long before the potential loss of any participant via refusal or difficulty locating the person, the research assistant plays a significant role in the individual’s continued participation. Consistent with our recruitment strategies, we treat participation as a collaborative endeavor. We are courteous, empathetic, and professional with our participants and adapt our assessment procedures as needed to maintain good rapport.

### 3.4. Technical Troubleshooting with Patients

All participants undergo EMA training with research staff prior to going home with the device. In this training, participants practice responding to EMA and familiarize themselves with the device. We also provide a short EMA manual that answers frequently asked questions and troubleshoots common device issues. As mentioned, we call patients weekly during the EMA phase to troubleshoot any problems. These calls are not intended to remind the patient to complete assessments, but instead are focused on addressing technical issues as needed. Before issuing the devices to patients, all study staff field-tested the EMA protocol to ensure that the devices would function properly during the one-month EMA phase. In doing so, technical issues that were idiosyncratic to the devices were identified and anticipated when troubleshooting with patients. We were able to identify internal and external issues that would compromise our device’s functionality over time, including hardware issues such as SD cards being misplaced, or internal issues, such as having the clock fail to keep the correct time. Some patients reported that the phone failed to issue all four surveys over the course of a day. However, some patients suggested that this may be attributed to not always carrying the phone on their person. We found that restarting the system daily allowed for optimal performance, preventing complications that could arise from older generation phones.

## 4. Conclusions

In this article, we reviewed the promises and challenges of using EMA to better understand post-hospital outcomes in patients diagnosed with psychotic-spectrum disorders. These patients are at high risk for relapse, rehospitalization, and many other negative outcomes such as suicide. Most previous studies assessing factors leading to rehospitalization in this population are limited by use of retrospective reporting that may be biased or fail to capture the most important time-varying and immediate contextual variables predicting these negative outcomes. EMA is a feasible tool for better understanding treatment-relevant behaviors, symptoms, and functioning in patients with psychosis. Our initial experience using EMA with this population suggests that there are many potential challenges using mobile devices with patients with severe mental illness. However, these challenges are manageable with reasonable modifications to the protocol and, thus far, patients appear to enjoy using the devices and find them acceptable as a way to share their experiences. Our hope is that the EMA data that we collect will prove useful for designing future personalized, ecological self-management interventions using these same mobile devices to improve treatment adherence and engagement in patients with psychosis following discharge from a psychiatric hospitalization.
